# Measuring Abnormal Brains: Building Normative Rules in Neuroimaging Using One-Class Support Vector Machines

**DOI:** 10.3389/fnins.2012.00178

**Published:** 2012-12-13

**Authors:** João Ricardo Sato, Jane Maryam Rondina, Janaina Mourão-Miranda

**Affiliations:** ^1^Center of Mathematics, Computation and Cognition, Universidade Federal do ABCSanto André, Brazil; ^2^Department of Computer Science, Centre for Computational Statistics and Machine Learning, University College LondonLondon, UK; ^3^Neuroimaging Laboratory, Department and Institute of Psychiatry, Faculty of Medicine, University of Säo PauloSäo Paulo, Brazil; ^4^Department of Neuroimaging, Centre for Neuroimaging Sciences, Institute of PsychiatryKing’s College, London, UK

**Keywords:** machine learning, SVM, one-class, neuroimaging, pattern recognition

## Abstract

Pattern recognition methods have demonstrated to be suitable analyses tools to handle the high dimensionality of neuroimaging data. However, most studies combining neuroimaging with pattern recognition methods focus on two-class classification problems, usually aiming to discriminate patients under a specific condition (e.g., Alzheimer’s disease) from healthy controls. In this perspective paper we highlight the potential of the one-class support vector machines (OC-SVM) as an unsupervised or exploratory approach that can be used to create normative rules in a multivariate sense. In contrast with the standard SVM that finds an optimal boundary separating two classes (discriminating boundary), the OC-SVM finds the boundary enclosing a specific class (characteristic boundary). If the OC-SVM is trained with patterns of healthy control subjects, the distance to the boundary can be interpreted as an abnormality score. This score might allow quantification of symptom severity or provide insights about subgroups of patients. We provide an intuitive description of basic concepts in one-class classification, the foundations of OC-SVM, current applications, and discuss how this tool can bring new insights to neuroimaging studies.

## Introduction

Several quantitative methods are available to analyze neuroimaging data. The development of voxel-based-morphometry (Ashburner and Friston, 2000), cortical surface modeling (Fischl et al., 1999), and deep-structures volumetry (Bigler et al., 1997; Appenzeller et al., 2005; Zetzsche et al., 2006)started a remarkable series of innovation. At the same time, functional magnetic resonance imaging (fMRI) based on BOLD signal (Ogawa et al., 1990) has become widely used in Neuroscience research. All these recent developments combined with advances in imaging acquisition protocols led to the accumulation of a huge amount of data.

There have been many applications of machine learning to clinical problems (e.g., classifying patients vs. healthy controls; Kloppel et al., 2011; Mwangi et al., 2012). However an important aspect that has been less explored is how to define normative rules for neuroimaging data of a population in order to define what a “typical brain” is and how to measure the distance from a single subject to these patterns of typical brains. Pattern recognition methods are highly suitable for this purpose, since they were developed to automatically discover regularities in high dimensional data through the use of computer algorithms (Bishop, 2006). To the best of our knowledge, there are no initiatives toward trying to define normative rules in Neuroimaging using other unsupervised learning methods. There is an inherent difficulty in evaluating results from this kind of methods (e.g., how to determine the ideal number or size of the clusters). Optimization is usually solved in a suboptimal manner using heuristic means, as an objective solution is either unknown or unfeasible due to algorithmic complexity and computational effort (Möller et al., 2010). Janoos et al. (2010) presented preliminary results from the application of a diffusion distance to perform clustering on the space of fMRI volumes in order to identify distinct brain states. Nettiksimmonsa et al. (2010) were able to identify subtypes among healthy controls that might represent the earliest stages of subclinical cognitive decline and Alzheimer’s disease using clustering. However, they relied on visual assessment for choosing the number of clusters.

In this perspective article, we aim to discuss how the machine-learning framework can be applied to build normative rules from neuroimaging databases. We propose the one-class support vector machines (OC-SVM) as a suitable tool for this purpose. We also discuss some technological challenges and perspectives regarding applications of OC-SVM to neuroimaging data.

## Pattern Recognition and Neuroimaging

Most of the studies applying pattern recognition methods to clinical neuroimaging focus on two-class classification problems, usually a group of healthy/typical subjects and a group under a very specific condition (e.g., major depression, Alzheimer’s disease, etc). In the general framework, pattern recognition approaches receive a set of observations (input variables and their respective class labels) and estimate a decision rule or model that can be applied to new observations. Once the rule is learned, given the input variables of a new example the model returns its expected label. So, the pattern recognition framework can be described as a machine that makes a class prediction *y* (label) for some unseen input vector *x* (input variables).

The most popular pattern recognition approach used in neuroimaging applications is the Support Vector Machine (SVM, Boser et al., 1992; Cortes and Vapnik, 1995; Vapnik, 1998). The SVM has received increasing interest due its attractive properties such as high generalization power (i.e., its ability to perform accurately on new, unseen examples after trained on a data set, which is usually called training data) and good scalability for high dimensional data, a particularly important property for neuroimaging applications. An additional important property that will be discussed in following sections is the SVM ability to perform non-linear classification (when the rule to obtain class prediction is based on a non-linear function of the input variables).

The fact that the neural substrates of some neurological/psychiatric disorders are very heterogeneous might limit the applications of two-class SVM to discriminate these patients’ populations from healthy subjects. Individuals suffering from a pathological condition can exhibit different patterns of brain abnormalities (e.g., subtypes), which will affect the ability of pattern recognition approaches to find a reproducible discriminating pattern.

## Normative Rules and Metrics

**Figure 1:**
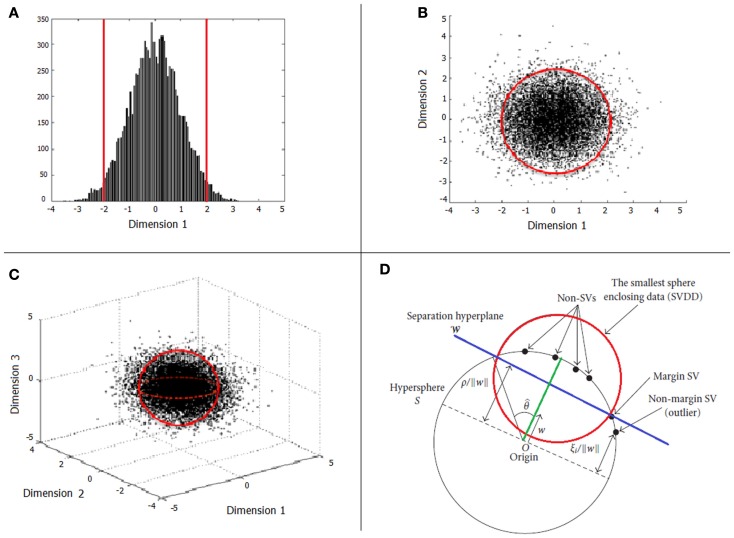
**Examples of boundary specification in the cases of one (A), two (B), and three (C) input variables**. Illustration of OC-SVM concepts are depicted in **(D)**.

When only one input variable is available (e.g., neuropsychological scale), an intuitive approach to define normative rules is finding a “normative” interval containing most of the typical observations. Thus, for an unseen observation, we may decide whether it is typical or not by checking whether it is within the normative interval (Figure 1A). When a specific percentage is defined, for example 95%, this interval is usually defined by the minimum length interval containing 95% of the typical observations (and thus, 5% of typical observations would be declared as atypical). When two input variables are considered, we may extend this approach to a bidimensional probability distribution, and define a normative boundary, which is the minimum area containing 95% of the typical observations. In Figure 1B, observations outside the red circle defining the boundary would be considered outliers. The same approach can also be extended to the case of three input variables (Figure 1C), but it is easy to see that the problem becomes more difficult when the number of dimensions increases to tens or hundreds. In case of high dimensionality, one may be tempted to define normative rules independently for each variable (i.e., in a mass-univariate approach). However, if these variables are correlated (which is usually the case in neuroimaging data), even though a new observation is declared as typical by all “univariate” rules, it can be atypical from a multivariate perspective, i.e., when jointly taking into account all dimensions. In this situation, the most appropriate approach would be to define “multivariate” normative rules.

When the number of input variables is small compared to the number of observations, it is possible to use the data to estimate a multivariate density function (Scott, 1992). Computing the probability density for neuroimaging data might become intractable due to its extremely high dimensionality (equal to the number of input variables derived from an image, possibly the number of intracranial voxels) and the relatively small sample sizes usually available (number of subjects). Alternatively, one can use methods that only find a decision boundary and do not rely on density estimation such as the OC-SVM. In fact, most of the SVM theory was based on Vapnik’s (1998) principle: “never to solve a problem which is more general than the one we actually need to solve.” Thus, if our aim is determining the minimum region (interval, area, etc.) containing a fixed percentage of the typical data we do not need to estimate the full density function of the referred multidimensional variable.

The challenge of defining normative multivariate rules is also known as data description problem in the machine-learning literature (Fayyad et al., 1996; Tax and Laskov, 2003). This problem can be also framed as an outlier or novelty detection approach where the aim is to detect uncharacteristic observations. A possible application of data description is to classification problems where one of the groups (or classes) is representatively sampled (e.g., large random samples), while the other group is severely under sampled. Unbalanced classes are very common in clinical setting where data from healthy subjects can be easier or less expensive to obtain than data from patients.

The OC-SVM computes a decision boundary with the minimal volume around a set of observations from the target group. Once the decision boundary is computed it can be used to classify new observations as outliers (if they fall outside the boundary) or not.

## Understanding the OC-SVM

### Basic concepts

One-class support vector machines is an unsupervised learning method proposed by Scholkopf et al. (2001). It focuses on determining a boundary enclosing the typical observations considering only one class of observations for training (called *target class*). After the training, new observations can be classified either as typical (in-class) or atypical (outliers), depending on their position in relation to the boundary.

Assume we have a training data *D* composed of *N* observations *x*_i_ (e.g., brain images) of dimension *d* (number of input variables), i.e., *D* = {*x*_1_, …, *x_N_*} in ${\mathbb{R}}^{\text{d}}$. *Our aim is to find the most compact region in* ${\mathbb{R}}^{\text{d}}$ *containing* most of the typical observations. The OC-SVM approach to solve this problem is to learn a mapping function from the input variables to a real number $\left(f_{x}:{\mathbb{R}}^{\text{d}}\to \mathbb{R}\right)$, such that most of data in *D* are mapped as positive values (the typical class) by *f_x_*. In other words, they belong to the set $R_{x}=\left\{x\in {\mathbb{R}}^{\text{d}}\text{ with }f_{x}\left(x\right)\geq 0\right\}$ while minimizing the volume of *R_x_*. This problem is called MVS (*minimum volume set*) estimation.

The OC-SVM belongs to the class of kernel methods. Kernel methods make use of kernel functions to find relationships or patterns in the data, which can be used to take actions such as classification (Shawe-Taylor and Cristianini, 2004). Kernel functions can be informally introduced as “similarity measures” to provide an intuitive understanding (Schölkopf and Smola, 2002). In the linear case this “similarity” can be expressed as the dot product between the input vectors representing the observations. A non-linear kernel corresponds to a mapping from the input variables (input space) to another space (feature space) using a non-linear transformation. A linear classification can then be carried out at this feature space and a linear boundary in the feature space corresponds to a non-linear boundary in the input *space*.

Formally a kernel function can be defined as a function that given two observations *x* and *x*′ ∈ *X* satisfies *k*(*x*, *x*′) = 〈Ø(*x*), Ø(*x*′)〉, where *X* is the input space or domain, Ø is a function mapping from *X* to a feature, and 〈 , 〉 is the dot product. Therefore non-linear kernels can be used to compute dot products in feature spaces without explicitly mapping the observations into the spaces, property commonly known as kernel trick.

A number of non-linear kernel functions have been proposed for kernel methods but the Gaussian or Radial Basis Function (RBF). Kernel is the most popular kernel function used with the OC-SVM. It depends on the Euclidian distance between the examples and is defined as $k\left(x,x^{\prime }\right)=e^{-{\left\|x-x^{\prime }\right\|}^{2}/2\gamma^{2}}$. The parameter γ can be thought as the distance used to measure the dissimilarity between the examples and it will influence the smoothness of the boundary (in the input space). This parameter is usually set using heuristics or tuned using cross-validation procedures (Scholkopf et al., 2001).

In Figure 1D we present an illustration of the *ν*-OC-SVM solution based on the RBF kernel. The target data (solid black circles) is mapped (through the RBF kernel) from the input variables space onto a hypersphere (in black). The problem then consists in finding the smaller hypersphere (in red) enclosing a pre-defined percentage of the observations [i.e., the (1 − *ν*)]. Note that this optimization problem can be reformulated as finding the most distant hyperplane (in blue, defined by a weight vector *w*) from the origin, such that (1 − *ν*) percent of the observations will be separated from it. In other words, the OC-SVM algorithm returns a function *f* that takes a positive value in the minimum region capturing most of the training data (typical observations) points and a negative value elsewhere (atypical). The OC-SVM can also be viewed as a two-class classification problem where the target data is one of the classes and the origin is the other.

In one-class classification problems, a false positive occurs when a true typical observation is erroneously classified as being atypical. For a given classifier, the probability of false positive misclassification is named as the false positive rate. An issue to be addressed in OC-SVM is the choice of the false positive rate parameters *ν*. The parameter *ν* can be fixed *a priori* and it corresponds to the percentage of observations of the typical data, which will be assigned as atypical (Type I Error).

### Measuring how typical a subject is

The classification in OC-SVM is based on the outcome of the decision function (*f_x_*), which in case of the RBF kernel is a non-linear function of the input variables. For a new observation, if this function value is positive, it means the observation is inside the boundary (“normative rule”) defining the typical examples. Otherwise, if the function value is negative it means the observation is outside the boundary and therefore is classified as atypical. Furthermore the relative position from the boundary can be used as a measure of abnormality (“normative metric”), which can then be correlated with other clinical or psychological measures for validation.

## Current Applications in Neuroimaging

There have been a few applications of the OC-SVM to non-clinical fMRI data. Hardoon and Manevitz (2005) applied OC-SVM to learn the pattern of brain activity associated with a motor task. In a proof of concept paper, Sato et al. (2009) applied the method to motor networks based on functional connectivity estimation in order to construct a normative connectivity database. This study has shown that subjects identified as outliers were scored at the tails of the distribution of a laterality index (Edinburg Inventory). Song and Wyrwicz (2009) applied the OC-SVM as an approach to classify voxels as activated or non-activated. According to the authors this framework can provide robust and accurate mapping of functional activation.

**Figure 2:**
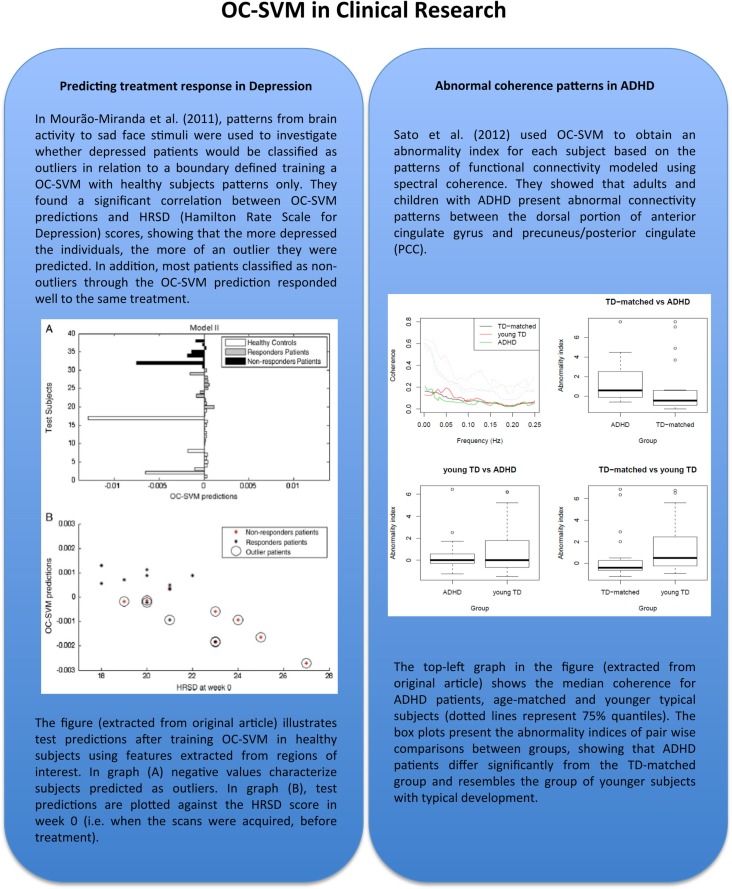
**In this figure-box we illustrate two applications of OC-SVM in studies involving different neuropsychological conditions: major depression disorder and attention-deficit/hyperactivity disorder (ADHD), both addressing the question of defining a boundary characterizing distributions of brain activation patterns from a normal population**. These papers are among the pioneer works representing important proof of concept that shows the potential of applying OC-SVM classifiers in order to obtain biomarkers for diagnosis or even prognosis in neuropsychological conditions. Although there are still few applications to date, this approach seems to be one of the trends in neuroimaging methods for clinical research with a high potential to be used in clinical routine in the near future. The figures were adapted and reproduced with the authorization of the original publishers.

Regarding clinical applications (see Figure 2), a recent work has demonstrated possible advantages of using OC-SVM with respect to two-class SVM in classifying depressed patients vs. healthy controls based on fMRI data. Fu et al. (2008) applied a standard two-class SVM to classify healthy controls vs. unipolar depressed patients based on the whole brain patterns of activation to an emotional stimulus (sad faces). However the authors were not able to predict treatment response with a significant accuracy as the number of responders and non-responders patients were not enough to train a two-class SVM. The same data set was later re-analyzed using the OC-SVM by Mourão-Miranda et al. (2011). In this study the authors found a significant correlation between the OC-SVM predictions and the patients’ Hamilton Rating Scale for Depression, i.e., the more depressed the patients were the higher their abnormality score was. Furthermore, the OC-SVM split the patient group into two subgroups whose memberships were associated with future response to treatment. This example illustrates the potential added value of using OC-SVM framework with respect to the two-class classification.

A more recent application of OC-SVM to neuroimaging data has been to investigate attention-deficit hyperactivity disorder (ADHD, Sato et al., 2012; see Figure 2). In this work, the authors have applied OC-SVM to patterns of functional connectivity and demonstrated that adults and children with ADHD present abnormal connectivity. An interesting conclusion was that the connectivity patterns of ADHD patients were more similar to the ones of younger typical developments subjects, in agreement with the hypothesis that the disorder is associated with abnormal brain maturation.

## Technological Challenges

It should be emphasized that the OC-SVM tries to solve a less constrained and therefore more difficult problem than the standard two-class SVM. In conventional two-class classification, in which the data from the two classes are available, the decision boundary is supported from both sides by training examples. In one-class classification only one class of data is available, and thus the boundary is only supported from one side. One of the challenges is to decide how tightly the boundary should fit in each of the dimensions around the data (defined by the kernel parameter). The curse of dimensionality becomes more severe in one-class classification problems as the boundary has to be defined in all directions. It is therefore expected that the one-class classification will require larger sample size for training in comparison with conventional classification.

Another obstacle, inherent to the unsupervised nature of OC-SVM, is the high susceptibility to noise and uninformative variables. OC-SVM attempts to take into account the entire set of input variables to learn common patterns. A direct consequence of this limitation is that the input variables should be carefully chosen when applying OC-SVM. Therefore priori knowledge about the relevance of input variables is necessary to increase the sensitivity of the method.

Finally, it is important to mention that the measure of abnormality obtained using OC-SVM is not specific, once this index reports deviation from normality in any direction. Thus, the labeling as an “outlier” does not provide any qualitative information about the abnormality, suggesting that further exploration of the data must be carried out.

## Future Directions

Considering the increase of large multi-center neuroimaging databases available we foresee also an increase on the applications of one-class classification approaches for defining normative rules based on these data. There are already some initiatives aiming to build large-scale neuroimaging databases such as the 1000 Connectomes (Biswal et al., 2010), ADNI (Mueller et al., 2005), and the ADHD-200 Sample^1^. In addition, there are some interdisciplinary and collaborative efforts to provide preprocessed data for both functional and structural neuroimaging^2^. The relevance of these initiatives in clinical applications is evidenced by the recent boom of studies using these shared databases (Babiloni et al., 2012; Meier et al., 2012). Public available databases are very attractive resources for the application of pattern recognition, machine learning, and data-mining methods as they normally consist of large number of subjects enabling the development and test of different models.

As we previously discussed, one of the main obstacles when dealing with structural/functional normative databases is how to define a “typical” brain pattern using high dimensional data. The high inter-subject variability and distinct etiologies of most neurological and neuropsychiatric conditions make the problem extremely difficult to tackle. The OC-SVM is a promising tool that can be applied to explore and extract relevant characteristics and information from these large databases with the aim of building normative rules. As in other medical areas, a cut-off value can be determined by locating a subject score in relation to the distribution derived from a number of healthy subjects. The fact that the OC-SVM approach can rely entirely on data from a healthy control sample makes it particularly suitable for the identification of rare disorders when only data from a very small number of patients are available or for the identification of subgroups of patients.

## Conflict of Interest Statement

The authors declare that the research was conducted in the absence of any commercial or financial relationships that could be construed as a potential conflict of interest.

## Footnotes

^1^http://fcon_1000.projects.nitrc.org/indi/adhd200/

^2^http://neurobureau.projects.nitrc.org/
